# *C. elegans* MANF Homolog Is Necessary for the Protection of Dopaminergic Neurons and ER Unfolded Protein Response

**DOI:** 10.3389/fnins.2018.00544

**Published:** 2018-08-10

**Authors:** Cory Richman, Sabih Rashid, Shreya Prashar, Ram Mishra, P. Ravi Selvaganapathy, Bhagwati P. Gupta

**Affiliations:** ^1^Department of Biology, McMaster University, Hamilton, ON, Canada; ^2^Department of Psychiatry and Behavioural Neuroscience, McMaster University, Hamilton, ON, Canada; ^3^Department of Mechanical Engineering, McMaster University, Hamilton, ON, Canada

**Keywords:** MANF, CDNF, *manf-1*, *C. elegans*, ER stress, dopamine, neurodegeneration, Parkinson's disease

## Abstract

Neurotrophic factors (NTFs) are important for the development, function, and survival of neurons in the mammalian system. Mesencephalic astrocyte-derived neurotrophic factor (MANF) and cerebral dopamine neurotrophic factor (CDNF) are two recently identified members of a novel family of NTFs in vertebrates that function to protect dopaminergic neurons. Although these genes are conserved across eukaryotes, their mechanism of neuroprotection is not fully understood. Sequence searches for MANF/CDNF homologs in invertebrates have identified a single ortholog that is most related to MANF. Here we report the *in vivo* characterization of the MANF gene, *manf-1*, in the nematode *Caenorhabditis elegans*. We found that *manf-1* mutants have an accelerated, age-dependent decline in the survival of dopaminergic neurons. The animals also show increased endoplasmic reticulum (ER) stress, as revealed by reporter gene expression analysis of *hsp-4*, an ER chaperone BiP/GRP78 homolog, suggesting that a failure to regulate the ER unfolded protein response (ER-UPR) may be a contributing factor to dopaminergic neurodegeneration. Expression studies of *manf-1* revealed that the gene is broadly expressed in a pattern that matches closely with *hsp-4*. Consistent with the requirements of *manf-1* in the ER-UPR, we found that aggregates of α-Synuclein, a major constituent of Lewy bodies, were significantly increased in body wall muscles of *manf-1* mutant animals. Overall, our work demonstrates the important role of *manf-1* in dopaminergic neuronal survival and the maintenance of ER homeostasis in *C. elegans*.

## Introduction

The nervous system controls all aspects of animal behavior by coordinating responses to internal and external stimuli. Because neurons are constantly processing signals and relaying information, failure to control physiological processes may compromise their ability to function normally and can contribute to neurodegeneration. Cellular programs that counteract harmful conditions such as the unfolded protein response (UPR) induced by mitochondrial and ER stresses protect neuronal cells by upregulating the expression of protein chaperones to promote proper protein folding and the maintenance of macromolecules.

During the development of both vertebrate and invertebrate nervous systems, excess neurons are pruned via programmed cell death in order to refine the enduring population of cells. Neurotrophic factors (NTFs) play an important role in this process (Levi-Montalcini and Angeletti, 1968). NTFs are secreted proteins capable of mediating a variety of neuronal signaling responses throughout development including growth, survival, differentiation, neurogenesis and synaptic plasticity. The best characterized NTFs comprise three major families: the neurotrophins, glial cell line-derived neurotrophic factor (GDNF) family of ligands (GFLs) and neuropoietic cytokines (Barbacid, 1995). More recently a novel family of NTFs has been identified that includes mesencephalic astrocyte derived neurotrophic factor (MANF) and its paralog cerebral dopamine neurotrophic factor (CDNF) (Petrova et al., 2003; Lindholm et al., 2007).

**Figure 1:**
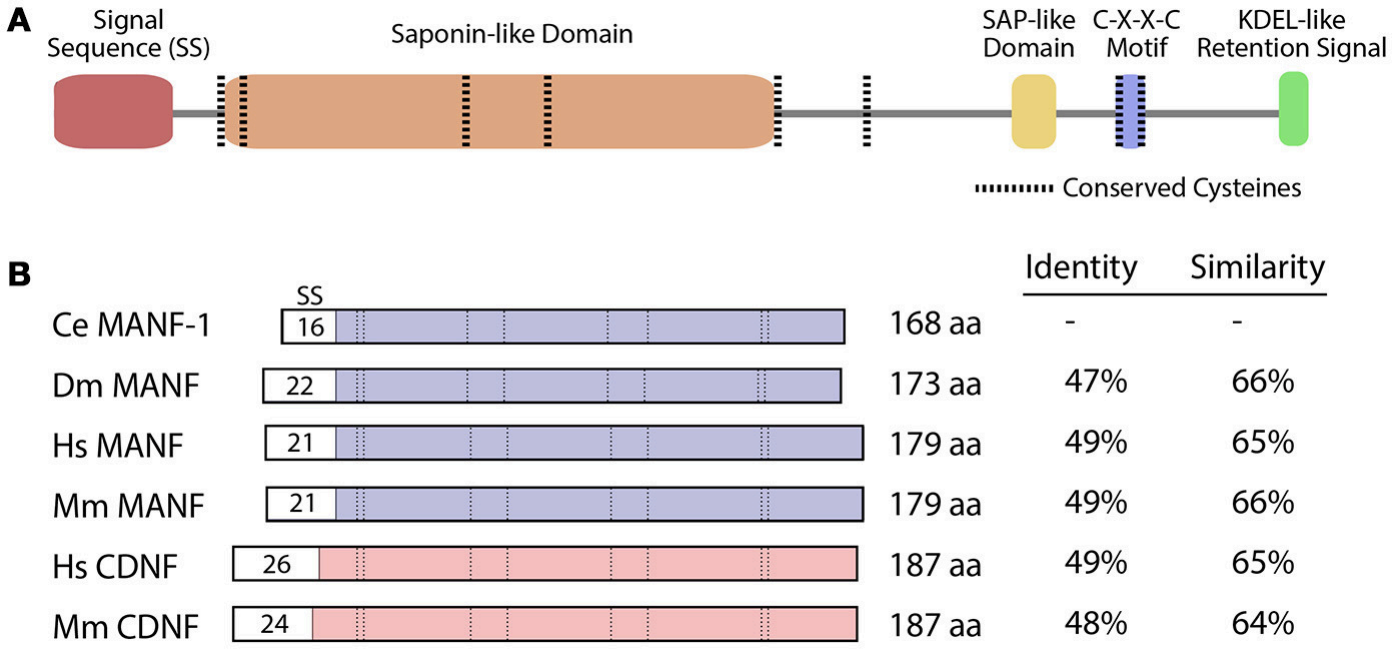
MANF and CDNF homologs in selected organisms. **(A)** Protein domains and structure of the *C. elegans manf-1* protein. **(B)** Schematic representation of MANF and CDNF proteins from *Caenorhabditis elegans (Ce), Drosophila melanogaster* (*Dm*), *Homo sapiens* (*Hs*) and *Mus musculus* (*Mm*) with percent identity and similarity indicated relative to *C. elegans manf-1*. Conserved domains are aligned and signal sequences (SS) are depicted with sizes, all presented to scale. GenBank accession numbers for amino acid sequences are described in Figure S1.

The structures of MANF and CDNF can be characterized as bifunctional, with neurotrophic activity believed to reside primarily within the N-terminal of the protein and the ER stress response within the C-terminal (Figure 1A; Parkash et al., 2009; Hellman et al., 2011). These proteins are composed of two globular alpha helical domains connected by a short linker, with eight highly conserved cysteines that form disulphide bonds in their mature conformations (Figure 1A; Mizobuchi et al., 2007; Parkash et al., 2009; Hoseki et al., 2010; Latge et al., 2015). The N-terminus is homologous to the Saposin protein superfamily, involved in the degradation of glycosphingolipids from the plasma membrane (Kolter and Sandhoff, 2005). It is the first Saposin-like protein (SAPLIP) structure shown to impart neuroprotective properties (Parkash et al., 2009; Hoseki et al., 2010; Hellman et al., 2011). The C-termini of MANF and CDNF contain putative retention signal sequences, RTDL and KTEL respectively, which closely resemble the canonical KDEL ER retention signal. This sequence is necessary and sufficient for the binding of MANF to KDEL receptors (Henderson et al., 2013). Sequence analysis has also revealed a C-X-X-C motif that is common to protein disulphide isomerases (PDIs). During periods of ER stress, PDIs can catalyze the formation of intramolecular disulphide bonds to restore proper protein folding (Ni and Lee, 2007).

MANF and CDNF proteins have been shown to protect dopaminergic neurons against a variety of insults, e.g., 6-hydroxydopamine (Airavaara et al., 2009; Voutilainen et al., 2009; Yu et al., 2010). Although the mechanism of neuroprotection is not well understood, both NTFs affect the ER-UPR and are likely to be important for the maintenance of ER homeostasis (Lee et al., 2003; Apostolou et al., 2008; Voutilainen et al., 2015). Elucidation of the molecular function of MANF and CDNF could lead to the development of targeted therapies for neurodegenerative disorders that are linked to ER stress such as Parkinson's disease (Lindholm et al., 2006; Hetz and Saxena, 2017).

The invertebrates *D. melanogaster* (fruit fly) and *Caenorhabditis elegans* (worm) possess a single MANF/CDNF homolog (Petrova et al., 2003; Lindholm et al., 2007), suggesting that the ancestral gene was duplicated in the vertebrate lineage (Figure S1). The *D. melanogaster* ortholog, *DmManf*, is necessary for the maintenance of dopaminergic neurites (Palgi et al., 2009), but is not required cell autonomously for their survival or differentiation (Stratoulias and Heino, 2015). *DmManf* is upregulated in response to ER stress, where it interacts with *Drosophila* homologs of mammalian UPR components including GRP78 (BiP), PERK, and XBP1 (Lindström et al., 2016). Despite the many similarities of MANF and CDNF, only human MANF is capable of rescuing the larval lethality associated with a *DmManf* knockout (Palgi et al., 2009). Very recently, a published study reported that the *C. elegans* MANF/CDNF homolog, *manf-1*, binds extracellular sulfatides (a class of glycosphingolipids) and enters the cell through endocytosis to mediate the ER stress response and confer cytoprotection (Bai et al., 2018). *C. elegans manf-1* mutants exhibit constitutive ER stress which involves the transduction proteins IRE-1 (ER stress sensor) and XBP-1 (ER stress activator) (Bai et al., 2018). Aside from the protein's activity in mitigating the ER-UPR, whether *manf-1* plays a specific role in the nervous system remains unknown.

We investigated the role of *manf-1* in neuroprotection using a combination of genetic and molecular approaches. The dopaminergic nervous system of *C. elegans* consists of two pairs of cephalic neurons (CEPs) and a single pair of anterior deirid neurons (ADEs) located in the head, along with another posterior deirid pair (PDEs) in the dorsal region of the body (Sulston et al., 1975). We examined the CEPs and ADEs in *C. elegans manf-1* mutants and observed that while these neurons were normal in 1 day old adults, degeneration was accelerated with age. We also found that the expression of the ER-UPR marker, *hsp-4p::GFP*, was upregulated in the absence of *manf-1* function, suggesting that this increase may contribute to the neurodegenerative phenotype. Reporter gene expression studies revealed that *manf-1* localization closely resembles that of the HSP-4 ER chaperone, further supporting *manf-1*'s role in the ER-UPR to confer protection to dopaminergic neurons. Since the abnormal accumulation of α-Synuclein, a major constituent of Lewy bodies, is linked to increased ER stress and dopaminergic neurodegeneration in vertebrates, we investigated the effect of *manf-1* on *SNCA (*α*-Synuclein)* gene expression. Examination of transgenic animals that express *SNCA::GFP* under the control of myosin heavy chain *unc-54* gene promoter revealed a significant increase in GFP fluorescent puncta in body wall muscles suggesting the enhanced aggregation of α-Synuclein in the absence of *manf-1* function. Overall, these results show that *manf-1* plays an important and conserved role in the dopaminergic nervous system of multicellular eukaryotes.

## Results

### Neuronal development is normal in *C. elegans manf-1* mutants

The Y54G2A.23 open reading frame in *C. elegans* encodes a protein that represents the closest homolog of vertebrate MANF and CDNF (http://www.wormbase.org), sharing conserved structural features including eight cysteines located at characteristic positions and a C-terminus ER-retention signal (Figure 1A; Petrova et al., 2003; Lindholm et al., 2007). The amino acid sequence alignment of Y54G2A.23 shows that it most closely resembles the MANF gene in *D. melanogaster* and vertebrate homologs (Figure 1B, Figure S2). Other nematode species also contain open reading frames with greater similarity to MANF compared to CDNF (http://www.wormbase.org). The mouse and human MANF proteins are roughly 65% similar to the *C. elegans* counterpart (Figure 1B).

**Figure 2:**
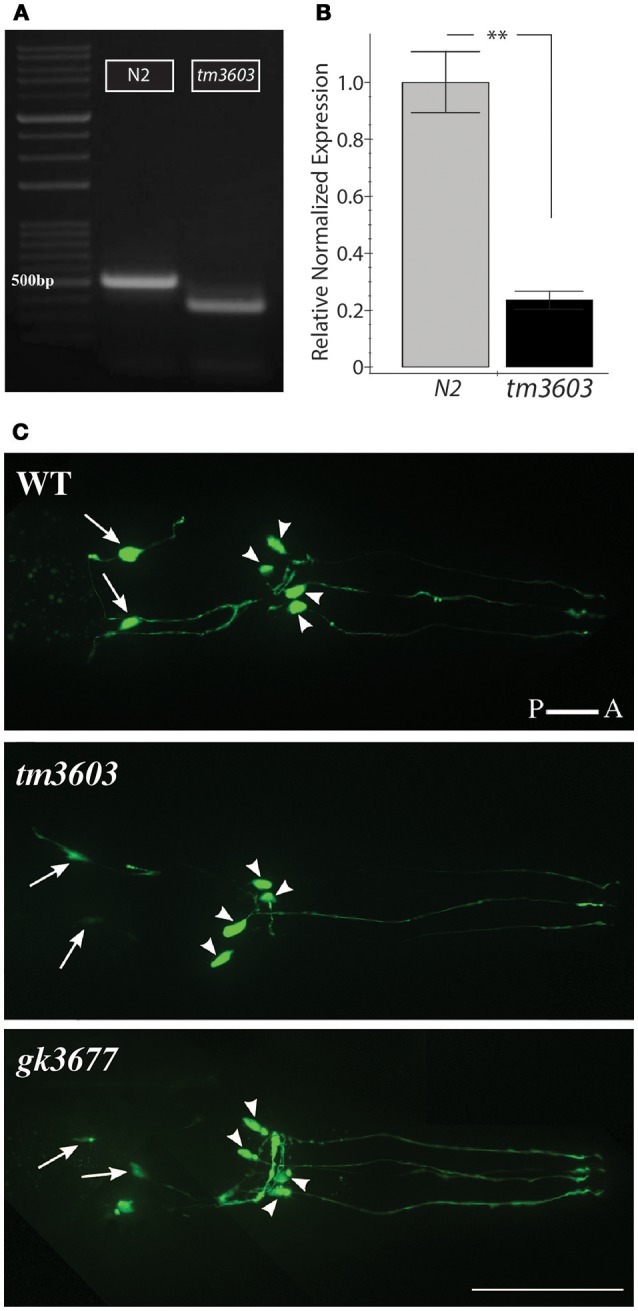
Characterization of *manf-1* alleles. **(A)** *manf-1* cDNA from wildtype (N2) and mutant (*tm3603*). 500 bp DNA ladder is marked for size comparison. **(B)** qRT-PCR analysis of *manf-1* in 1 day old adults. Results are means of experiments performed in triplicate ± SEMs, **p < 0.01. **(C)** Visualization of dopaminergic neurons in the head of wildtype (WT), *tm3603* and *gk3677* mutant alleles. The posterior pair of ADEs is indicated by arrows and four anterior CEPs are indicated by arrowheads. Scale bar = 50 μm.

A deletion allele of *manf-1, tm3603* (National BioResource Project, https://shigen.nig.ac.jp/c.elegans), removed the third exon which we confirmed by sequencing. The allele carries a 204 bp long deletion (flanking 30 nucleotides: TCTAATTTTCCTTTAAAATTTTTAATTTTT and TAAATCCCCAAATTTCACAGACAAGCCACT) and a random 21 bp insertion in place of the missing sequence. The mutation removes three conserved cysteines, as well as nearly half of the highly folded N' terminal Saponin-like domain. The majority of surface residues believed to impart the functional neuroprotective activity to human MANF are lost in the *tm3603* mutation (Parkash et al., 2009). Interestingly, cDNA analysis revealed the presence of a truncated mRNA transcript in *manf-1(tm3603)* worms indicating that read-through transcription occurs despite the presence of three in-frame nonsense mutations (Figure 2A, Figure S3). The truncated *tm3603* transcript is expressed at a low level as determined by qRT-PCR (Figure 2B). We do not know whether the *tm3603* allele is translated, although any such product is unlikely to be biologically functional. More recently, we also obtained a CRISPR allele of *manf-1, gk3677* (see Methods) that deletes the entire *manf-1* open reading frame. Both *tm3603* and *gk3677* animals are homozygous viable and appear healthy. This is strikingly different from *D. melanogaster DmManf* mutants, which exhibit early stage lethality (Palgi et al., 2009).

**Figure 3:**
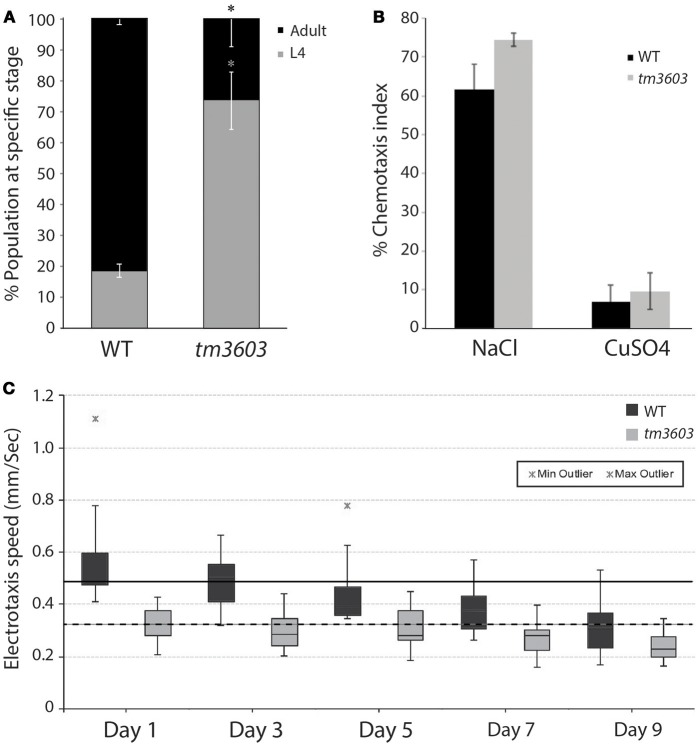
Chemosensory and life traits of *manf-1(tm3603)* animals**. (A)** Growth delay represented by percentage of animals reaching adulthood by 54 h after plating synchronized L1 larvae. Experiment performed in duplicate ± SEM, *p = 0.0144. **(B)** Results of chemotaxis assays for NaCl (chemo-attractant) and CuSO_4_ (chemo-repellent). Means are derived from four batches ± SEMs, demonstrating a functional and comparable chemotactic response between mutant and wildtype animals. **(C)** Box plot of the electrotaxis response of *tm3603* and wildtype (N2) controls on 1, 3, 5, 7, and 9 days of adulthood. The horizontal line inside each box marks the median, with upper and lower ends of boxes representing 25 and 75th quartile of data samples. Each vertical line with caps shows the spread of data. Stars indicate outliers. The speed of animals declines with age resulting in a 37% reduction in wildtype by day-9 and 30% in *tm3603* over the same period. The solid and dotted black horizontal lines serve as references for the median speeds of day-1 wildtype and *tm3603* animals, respectively. *p* < 0.01 for all days compared.

Visual inspection of *manf-1* mutants showed no obvious morphological defects, although the animals exhibited a significantly slower growth rate compared to wildtype (Figure 3A). The growth delay did not appear to affect adults as they looked healthy and had a normal lifespan (Figure S4). Next, we used three different reporter strains to examine major neuronal cell types in *tm3603* and *gk3677* animals. The *dat-1* (dopamine transporter) promoter-driven YFP reporter revealed normal dopaminergic neurite trajectories and number of soma at day-1 of adulthood (Figure 2C), suggesting that developmental processes were normal. Likewise, GABAergic and serotonergic markers (*unc-47*p::GFP and *tph-1*p::GFP, respectively) showed no obvious defects (Figure S5). We also examined the chemotactic phenotype of mutants. The chemosensory response is controlled by neurons receiving olfactory and gustatory cues and is mediated by a large portion of the nervous system (Bargmann, 2006). The *tm3603* animals were able to mount a normal chemotactic response to an attractant (NaCl) and a repellent (CuSO4) (Figure 3B). Together, these results suggest that *manf-1* does not play a major role in the formation and guidance of neurons in *C. elegans*.

### Neurodegeneration and ER stress responses are enhanced in *manf-1* mutant adults

**Figure 4:**
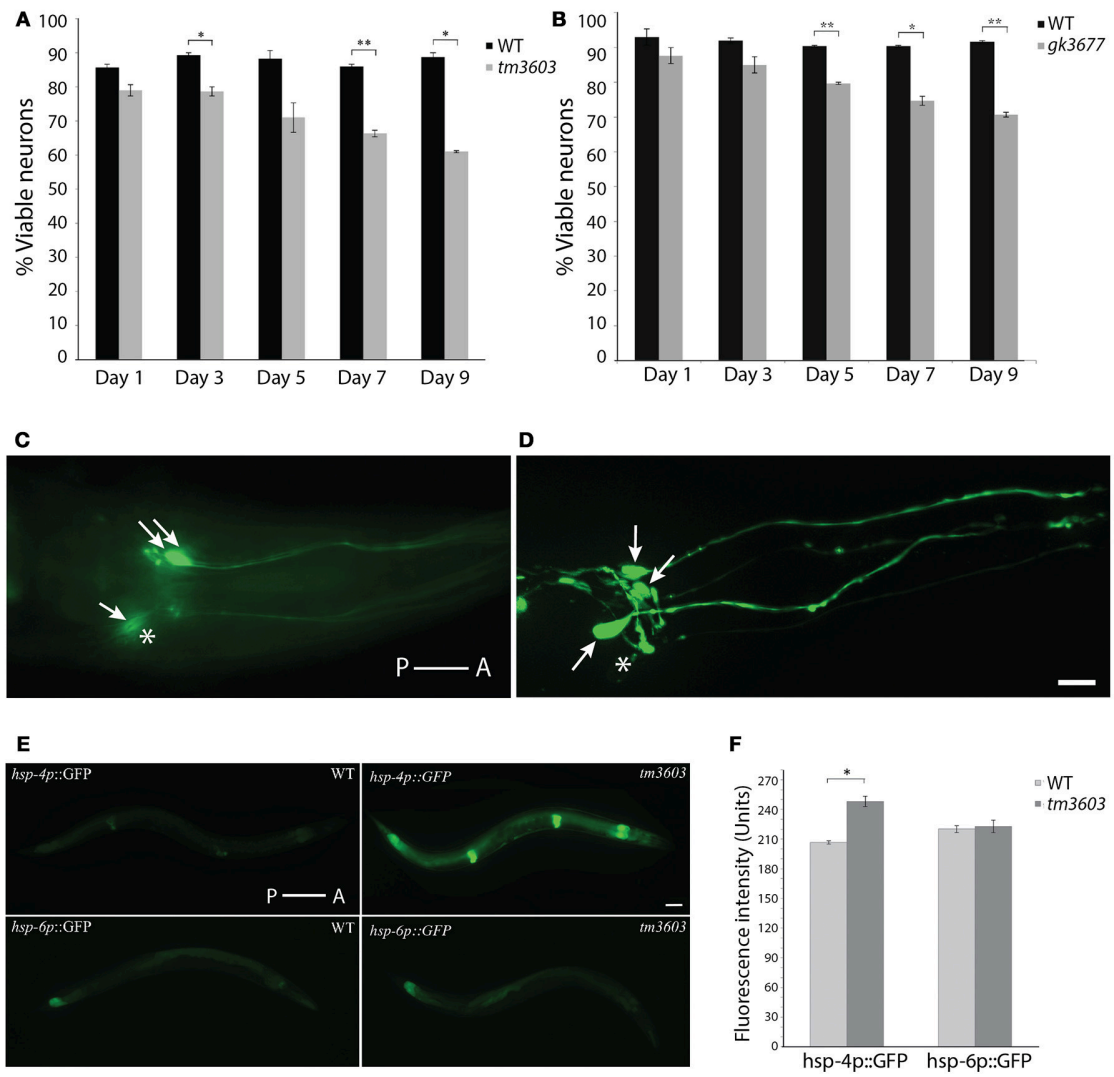
Dopaminergic neuron and ER stress phenotypes of *manf-1* mutants. Quantification of dopaminergic neuronal defects in day-1 to day-9 old adults of *tm3603* **(A)** and *gk3677* **(B)** mutant animals. In each case wildtype (WT) animals were used as controls. Experiments performed in duplicate ± SEMs, *p < 0.05, **p < 0.01. **(C,D)** Nomarski fluorescence **(C)** and Confocal **(D)** images of dopaminergic neurons in two different *tm3603* day-7 old animals. Arrows mark neuronal cell bodies that are visible and stars placed next to those that are faint and/or undetectable. Scale bar = 25 μm. **(E)** Differential induction of ER and mitochondrial stress. Whole animal ER (*hsp-4p*::GFP) and mitochondrial (*hsp-6p*::GFP) reporters visualized in wildtype and *tm3603* animals; worms with average fluorescence shown. Scale bar = 50 μm. **(F)** The average GFP pixel intensity values quantified via whole animal analysis, performed in triplicate ± SEMs, *p < 0.05. Anterior (A) and posterior (P) orientations in **(C–E)** are indicated.

Although neuronal development appears to be unaffected in *manf-1* mutants, the gene may have a neuroprotective role in adults. To investigate this putative neuroprotection, we examined the dopaminergic neurons of older animals. The cell bodies of ADEs and CEPs were counted and the appearance of dendritic projections were assessed on alternating days from the first until the ninth day of adulthood. We observed significantly enhanced degeneration in both *tm3603* and *gk3677* mutants by day-3, a phenotype that became progressively worse with age, with day-9 adults losing roughly one-third of the dopaminergic somas compared to wildtype controls (Figures 4A–D). Dendritic morphologies of both mutants were not remarkably different from controls. These results show that the presence of *manf-1* prevents the degeneration of dopaminergic neurons. We also examined serotonergic and GABAergic neurons in older adults (day-5 to day-7, *n* ≥ 15 for each genotype) but did not observe an obvious increase in degenerative phenotypes in *tm3603* animals. Some cases of ectopic branching and fused processes of GABAergic neurons were observed in both control and mutant worms (Figure S5). However, the interpretation and relevance of these changes will require further investigation.

Studies in different animal models have shown that the cytoprotective roles of MANF and CDNF may in part involve the ER-UPR (Voutilainen et al., 2015). Although both NTFs are secreted, they localize largely within the ER and may facilitate protein folding. *C. elegans manf-1* appears to play a similar role as examined by the GFP reporter expression analysis of *hsp-4*, a homolog of the human ER chaperone BiP/GRP78 that acts as a reliable ER stress response marker (Calfon et al., 2002). Evidence of this comes from the analysis of *manf-1(tm3603); hsp-4p*::GFP animals that showed a significant increase in GFP fluorescence which was particularly enhanced within the intestine, hypodermis and spermatheca (17% higher relative to controls, *P* < 0.05) (Figures 4E,F). Thus, *manf-1* appears to be necessary in maintaining the normal ER-UPR response within worms. We also examined *hsp-6* expression, an ortholog of the vertebrate HSP70 mitochondrial matrix specific chaperone (Yoneda et al., 2004), in *manf-1(tm3603)* animals using a *hsp-6p*::GFP reporter strain. No obvious change in GFP fluorescence was observed (Figures 4E,F), suggesting that *manf-1* may not play a role in mitochondrial UPR regulation.

We next investigated whether *manf-1* mutants have behavioral defects that might be attributed to neuronal and ER stress abnormalities. For this, we analyzed the electrotactic response of animals. Our lab has previously shown that such a response depends on an intact dopaminergic nervous system and functional dopamine signaling (Salam et al., 2013) (S. Salam and B. Gupta, unpublished). The electrotaxis assay revealed that *tm3603* adults had a considerably slower swimming speed compared to wildtype controls for the entire duration of testing, i.e., on 1, 3, 5, 7, and 9 days of adulthood (Figure 3C). The median speed was reduced by about 30% on all days tested (day-1 adults: 487 μm/s for wildtype and 324 μm/s for *tm3603*; day-9 adults: 306 μm/s for wildtype and 226 μm/s for *tm3603*). Although the responses did not decline further with age, possibly because neuronal signaling was compromised well before morphological changes were detectable in our GFP-based assay, the results show that *manf-1* is needed to maintain normal electrotactic behavior in adult worms.

### *manf-1* is widely expressed during development and its expression declines in adults

**Figure 5:**
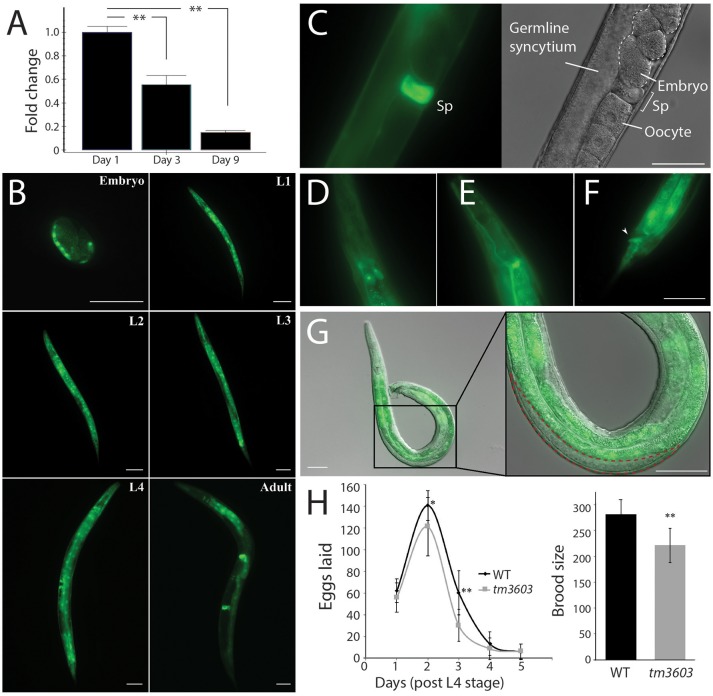
Expression profile of *manf-1* and fecundity defects in *manf-1* mutants. **(A)** qRT-PCR data showing a decline in endogenous *manf-1* transcript levels with age in wildtype animals. Results are means of experiments performed in duplicate ± SEMs, normalized to day-1 adult *manf-1* expression levels, **p < 0.01. **(B)** GFP fluorescence in *manf-1p::GFP* animals at different stages of development (embryo, larval L1 to L4, and adult). **(C)** Bright fluorescence within the spermatheca (Sp) of adults. **(D–F)** Expression within the excretory system; excretory gland cells **(D)**, canal **(E)**, and anus **(F)** indicated by arrowhead. **(G)** Gonad of an adult *manf-1p::GFP* transgenic male showing a lack of fluorescence in the region containing sperm (indicated by red dotted line). **(H)** Frequency of egg laying and brood sizes in wildtype and *tm3603* animals. Means ± SDs are plotted. *p < 0.05, ** < 0.01. Scale bar for all images = 50 μm.

The neurodegenerative phenotype of *manf-1* mutants, along with growth delay and increased ER stress prompted us to investigate the protein's expression pattern. To this end we initially used the SPELL search engine to query known transcriptome datasets (http://spell.caltech.edu:3000) (Hibbs et al., 2007). *manf-1* is expressed throughout developmental and post-developmental stages, declining with age to about 50% by day-8 of adulthood and beyond (Figure S6; Golden et al., 2008; Levin et al., 2012; Grun et al., 2014; Hashimshony et al., 2015). The age-dependent changes *in manf-1* transcription were confirmed by qRT-PCR. Specifically, we observed transcript levels to be lowered by roughly 55% by day-3 and 85% by day-9 of adulthood (compared to day-1 as a normalized control, Figure 5A).

To examine tissue-specific expression, we generated transgenic strains expressing a fluorescent reporter. A transcriptional reporter plasmid was constructed by placing GFP under the control of a 2.7 kb 5′-UTR of *manf-1*. Bright fluorescence was detected in post-gastrulating embryos in regions corresponding to intestinal cells and the dorsal body wall muscle quadrant (Figure 5B). Expression continued throughout all larval stages and into adulthood in most tissues except for the gonadal cells. By mid L4, GFP fluorescence was visible in intestinal and muscle cells, with concentrated regions within the pharyngeal and vulval muscles. There was an obvious change in the expression pattern of young adults as fluorescence decreased in the anterior head and posterior tail regions, but the intestine, pharynx and particularly the spermatheca (Figure 5C) continued to exhibit bright reporter expression. Fluorescence was equally observed in the excretory system within structures resembling the excretory gland cells, the intestinal-rectal valve and anus (Figures 5D–F). Such a broad domain of *manf-1* expression aligns well with the observed systemic increase in ER stress (based on *hsp-4p::GFP* patterns) in mutant animals and suggests that *manf-1* is likely to have additional roles in *C. elegans* outside of the dopaminergic nervous system, a finding consistent with reports attributing MANF function to other tissues (Mizobuchi et al., 2007; Lindholm et al., 2008; Tadimalla et al., 2008; Yu et al., 2010; Bai et al., 2018).

The bright fluorescence in the spermatheca may be analogous to MANF expression within the testes of mammals (Lindholm et al., 2008). Interestingly, no expression was observed in the male gonad (Figure 5G), suggesting sex-specific differences in the mechanism of *manf-1* expression. To determine whether *manf-1* accumulation in the spermatheca may have a role in reproduction, we examined the brood size of hermaphrodites. The reproductive span of *tm3603* animals was comparable to wildtype, however the overall brood size was lower due to fewer fertilized eggs being laid each day (Figure 5H). There is a notable decline in *manf-1* mutant progeny which is in line with previous evidence demonstrating a reduction in mutant offspring resulting from the inability to mount a proper ER stress response (Bai et al., 2018). Thus, *manf-1* plays a role in fertility although its mechanism of action remains to be determined.

### Loss of *manf-1* enhances the expression and aggregation of α-synuclein

Accumulating evidence has implicated ER stress in a wide array of neurodegenerative disorders which can lead to the build-up of misfolded proteins and disrupted calcium homeostasis. The impaired clearance of these protein aggregates triggers the ER-UPR, up-regulating genes (e.g., chaperone proteins) to re-establish homeostasis (Lindholm et al., 2006). A known hallmark of Parkinson's disease is the presence of intracellular protein inclusions within neurons known as Lewy bodies (Kalia and Lang, 2015). The aggregation of α-Synuclein, a major constituent of Lewy bodies, is enhanced by ER stress (Jiang et al., 2010).

**Figure 6:**
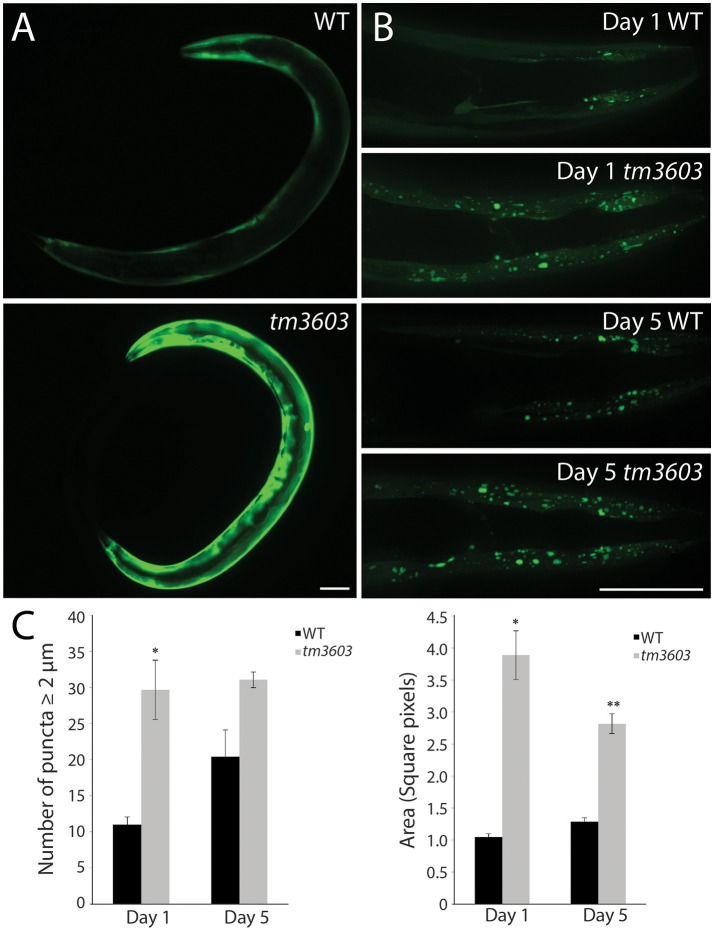
*manf-1* mutants show enhanced aggregation of α-Synuclein. **(A)** Expression analysis of human *SNCA::YFP* gene within the body wall muscles of wildtype (N2) and *tm3603* animals. **(B)** Maximum projection of Z-stack images at days 1 and 5 of adulthood showing YFP fluorescent puncta. **(C)** Number of puncta of diameter 2 μm and above determined by manual counting and automated analysis of total pixel area after thresholding. Experiments performed in triplicate ± SEMs, *p < 0.05, ** < 0.01. Scale bar = 50 μm.

To determine whether increased degeneration of dopaminergic neurons and ER stress in *manf-1* mutants leads to the accumulation of α-Synuclein, we investigated the formation of α-Synuclein clusters in transgenic animals using a YFP fluorescent reporter. The transgenic animals express a *YFP* reporter fused to human α-Synuclein under the control of the *unc-54* promotor for the visualization of protein clusters within the body wall muscle cells (Hamamichi et al., 2008; Van Ham et al., 2008). There was a significant increase in YFP fluorescence in 1 day old *tm3603* adults as well as number of inclusions (Figure 6). The accumulation of chimeric proteins as fluorescent puncta was easily visualized using fluorescence microscopy. Normally, the size of puncta in 1 day old wildtype animals is small; however, *manf-1* mutants had much brighter fluorescence (Figure 6A) and larger, more numerous inclusions (2 μm diameter and larger) within the head region compared to the wildtype control (Figures 6B,C). The trend was equally pronounced in 5 day old adults. Interestingly, the data does not show an age-dependent increase in the number and area of puncta in mutants. More work such as measuring and plotting individual puncta sizes (not just 2 μm threshold) as well as puncta analysis at later stages of adulthood, beyond day-5, are needed to resolve this matter. Overall, we conclude that *manf-1* is important for the normal expression and appropriate subcellular localization of α-Synuclein in *C. elegans*.

## Discussion

MANF and CDNF represent a novel class of NTFs that are important for the maintenance of the ER-UPR and protection of dopaminergic neurons in various animal models. In this study, we describe the role of the *C. elegans* MANF ortholog, *manf-1*, in cellular processes. While a complete removal of *manf-1* function showed no obvious morphological defects in young 1 day old adults, the animals were found to have a slight but significant growth delay during development. Examination of dopaminergic neurons in older *manf-1* mutant adults revealed accelerated degeneration with age, which suggests an essential role of *manf-1* in neuroprotection.

The transcriptional profile of *manf-1* revealed expression throughout developmental and post-developmental stages. Similar results were obtained in *manf-1p::GFP* transgenic animals. Careful analysis of GFP fluorescence in larval and adult stages showed that *manf-1* is expressed in many tissues including the intestine, muscle, spermatheca, and excretory system. The presence of GFP in spermathecal cells suggests that *manf-1* plays a role in reproduction, which is supported by the low brood size of mutants. We also observed a significant induction of ER stress in animals lacking *manf-1* function as determined by *hsp-4* promoter-driven reporter gene expression. Thus, *manf-1* is necessary to maintain the resting state of the ER-UPR. This finding is consistent with earlier results showing higher sensitivity of *manf-1* mutants to tunicamycin, a protein glycosylation inhibitor, causing a greater reduction in brood size compared to controls (Bai et al., 2018).

The broad similarity in *manf-1p::GFP* and *hsp-4p::GFP* expression in adult animals suggests the possibility of a shared mechanism of gene regulation. The promoter regions of mammalian MANF and UPR-targeted genes that encode ER-resident molecular chaperones such as BiP/GRP78 have been shown to possess an ER stress response regulatory element (ERSEII) (Kokame et al., 2001; Mizobuchi et al., 2007). This conserved element within the *manf* promoter appears to activate gene expression in a spatial and temporal manner that matches with BiP/GRP78 (Mizobuchi et al., 2007). Furthermore, this motif is unique to MANF and is not found within the CDNF promoter (Mizobuchi et al., 2007). Upon induction of the UPR, regulatory transcription factors bind to ERSEII sequences to promote the expression of chaperone genes. The *C. elegans manf-1* promoter shows partial matches to the ERSEII motif (C. Richman, unpublished), and so characterization of *manf-1* regulatory sequences in the future holds the potential to unravel conserved mechanisms of MANF regulation in the ER-UPR response.

The ER is a major site for protein synthesis and homeostasis. Perturbations in ER function due to disease and altered environmental conditions can result in the accumulation of aberrant proteins such as α-Synuclein, the main constituent of Lewy bodies in the brains of patients with Parkinson's disease (Kalia and Lang, 2015). Both MANF and CDNF have previously been shown to be neuroprotective against the toxicity induced by α-Synuclein oligomers, with MANF believed to mediate its protective response via the upregulation of BiP/GRP78 (Latge et al., 2015; Huang et al., 2016). As expected, we noted a significant increase in α-Synuclein aggregates in *manf-1* mutant animals. While more work is needed to determine whether *manf-1* plays a direct role in this process, the results reveal the important role of the protein in maintaining normal ER function and neuroprotection.

Identification of the *C. elegans manf-1* gene and its role in dopaminergic neurons provides a unique opportunity to explore the conserved mechanism by which the MANF/CDNF family of NTFs functions in eukaryotes. Determining precisely how MANF regulates components of the ER-UPR and MANF-interacting proteins will help direct the investigation of signaling mechanisms in neuroprotection. Additionally, this model may serve as a powerful screening tool for new therapeutics targeting MANF pathways.

## Materials and methods

### Strains and culture conditions

*Caenorhabditis elegans* worms were cultured on standard NG-agar plates using established protocols and *Escherichia coli* strain OP50 as a food source (Brenner, 1974). The strains were maintained at 20°C. Age-synchronized cultures were obtained by treating gravid hermaphrodites with sodium hypochlorite (3:2 ratio of NaOCl:NaOH) and transferring eggs onto new plates. Following is the list of strains used in this study.

Wildtype N2
DY353 *bhEx138[pGLC72(Cel-dat-1p::YFP)]*
DY487 *manf-1(tm3603)*
DY581 *manf-1(tm3603); zcIs4(hsp-4p::GFP)*
DY597 *manf-1(tm3603); bhEx247[pGLC72(Cel-dat-1p::YFP)]*
DY598 *manf-1(tm3603); zcIs13(hsp-6p::GFP)*
DY612 *bhEx259[pGLC135(Cel-manf-1p::GFP)*+ *pRF4(rol-6(su1006))]*
DY613 *manf-1(tm3603); mgIs42[tph-1p::GFP* + *pRF4(rol-6(su1006))]*
DY623 *manf-1(tm3603); oxIs12[unc-47p::GFP* + *lin-15(*+*)]*
DY658 *manf-1(tm3603); pkIs2386[unc-54p::SNCA::YFP* + *unc-119(*+*)]*
EG1285 *oxIs12[unc-47p::GFP* + *lin-15(*+*)]*
GR1366 *mgIs42[tph-1p::GFP* + *rol-6(su1006)]*
NL5901 *pkIs2386[unc-54p::SNCA::YFP* + *unc-119(*+*)]*
SJ4005 *zcIs4[hsp-4p::GFP]*
SJ4100 *zcIs13[hsp-6p::GFP]*
VC3705 *manf-1(gk3677)*

Some of the strains were obtained from the Caenorhabditis Genetics Center. Two *manf-1* mutant alleles, *tm3603* and *gk3677*, were kindly provided by the laboratories of Drs. Mitani (Tokyo Women's Medical University) and Moerman (University of British Columbia).

Egg laying and brood size counts were determined by cloning synchronized L4 stage nematodes and determining the sum of total eggs laid over a period of 5 days. Twenty animals were used per strain, and worms were transferred to fresh plates daily. Outliers indicative of sick animals or contamination were excluded. Growth delay between strains was determined by assessing the percentage of worms that reached adulthood by 54 h post-L1 arrest grown at 20°C via random sampling.

### Molecular biology and transgenics

The *manf-1p::GFP* reporter plasmid (pGL135) was generated by subcloning a *Pst*I, *BamH*I-digested PCR-amplified 2,755 bp 5′UTR fragment of *C. elegans manf-1* (using primers GL1123/GL1126) into the Fire lab vector pPD95.69. Transgenic strains were generated via microinjection (Mello et al., 1991).

### Quantitative RT-PCR assays

Total RNA was extracted from animals using the TRIZOL method. All samples were DNase treated (Thermo Scientific DNase I, cat. #EN0521) prior to preparing cDNA (NEB AMV First Strand cDNA Synthesis Kit, cat #E6550S). Reaction mixes for qRT-PCR were prepared using a SensiFAST SYBR Hi-ROX Kit (Bioline, cat #BIO-92005) on a Bio-Rad CFX96 Touch Real-Time PCR Detection System. *manf-1* was amplified using the primer pair GL916/GL1232. Experiments were normalized to the reference gene *pmp-*3. Data was generated and analyzed using BioRad CFX manager software 3.1.

Endogenous *manf-1* transcripts in wildtype animals were measured using a custom RT2 qPCR Primer Assay (Qiagen, cat #330001-PPL05030A), which included pre-designed optimized primers. Gene expression data was obtained on day-3 and day-9 of adulthood and normalized to day-1.

### Microscopy

Animals were mounted on 2% agar pads and anesthetized using 30 mM sodium azide. GFP fluorescence was visualized using a Zeiss Observer Z1 microscope equipped with an Apotome 2 and X-Cite® 120LED fluorescence illuminator. Neurodegeneration was manually scored by counting the number of cell bodies and by assessing neurite morphologies and trajectories. Samples were analyzed in duplicates with *n* = 25–30 per genotype per experiment.

Whole animal fluorescence was quantified in *hsp-4p::GFP* and *hsp-6p::GFP* reporter lines using a Nikon Eclipse 80i microscope equipped with a mercury lamp power supply (C-SHG1) and Hamamatsu ORCA-ER digital camera (C4742-80). Images were processed using NIH ImageJ (rsbweb.nih.gov/ij) software. Samples were analyzed in triplicate with *n* = 30 per genotype per experiment.

### Analysis of α-synuclein aggregation

To quantify α-Synuclein inclusions, an optimal exposure rate for each animal was individually determined from the brightest and most densely aggregated plane. Image acquisition parameters were fixed to 15 Z-stacks of varied slice intervals, accounting for variations in animal size and ensuring unbiased imaging of planes. The maximum projection of each Z-stack set was generated using Zen2 blue edition software (zeiss.com/microscopy/int/software-cameras.html), with the first and last image being determined by the furthest plane in each respective direction containing clear inclusions. Foci with a minimum diameter of 2 μm from the nose to posterior pharyngeal bulb were analyzed and manually counted. Automated quantification of inclusions was performed by calculating the total pixel area of fluorescence after thresholding images to eliminate background using ImageJ software (Salam et al., 2013). Samples were analyzed in triplicate with *n* = 30 per genotype per experiment.

### Statistical analysis

Data is presented as means ± SEM unless otherwise specified, and two-tailed *P*-values were calculated using unpaired *t*-tests with values <0.05 considered statistically significant. The values are indicated in figures where difference is relevant.

## Author contributions

CR, SR, and SP performed experiments. RM and PS provided input on some of the approaches. CR and BG analyzed data and co-wrote the manuscript. All authors participated in editing the drafts. BG designed and supervised the entire study.

### Conflict of interest statement

The authors declare that the research was conducted in the absence of any commercial or financial relationships that could be construed as a potential conflict of interest.
