# Supplementary material for: C. elegans MANF Homolog Is Necessary for the Protection of Dopaminergic Neurons and ER Unfolded Protein Response
Source: Front Neurosci. 2018 Aug 10;12:544. doi: 10.3389/fnins.2018.00544 (PMC6095968; doi:10.3389/fnins.2018.00544)
Supplement: Supplementary file 1 [file Data_Sheet_1.pdf]

SUPPLEMENTARY DATA

Cory Richman et al. "*C. elegans* MANF homolog is necessary for the protection of dopaminergic neurons and ER Unfolded Protein Response."

Figure S1. Phylogenetic analysis for selected MANF and CDNF full-length amino acid sequences. The tree was constructed using *MEGA7* (Kumar et al., 2016). Peptide sequences were aligned using ClustalW and evolutionary history was inferred using the maximum likelihood method based on the Le_Gascuel_2008 model (Le and Gascuel, 2008;Le et al., 2008) with substitution rates determined using the invariant and discrete gamma distribution models. The tree branches are drawn to scale, with lengths measured in the number of substitutions per site and bootstrap values indicated at branch points. GenBank accession numbers for each of the amino acid sequences used in the alignment (from top to bottom, with the species names preceding in italics) include: **CDNF** *H.s* NP_001025125.2; *P.t* XP_507666.3; *M.m* NP_808315.1; *R.n* NP_001032632.1; **MANF** *H.s* NP_006001.4; *P.t* JAA43993.1; *M.m* NP_083379.2; *R.n* P0C5H9.1; *X.t* NP_001016425.1; *D.r* NP_001070097.1; *D.m* NP_477445.1; *C.e* Q9N3B0.2; *C.b* CAP32313.1.

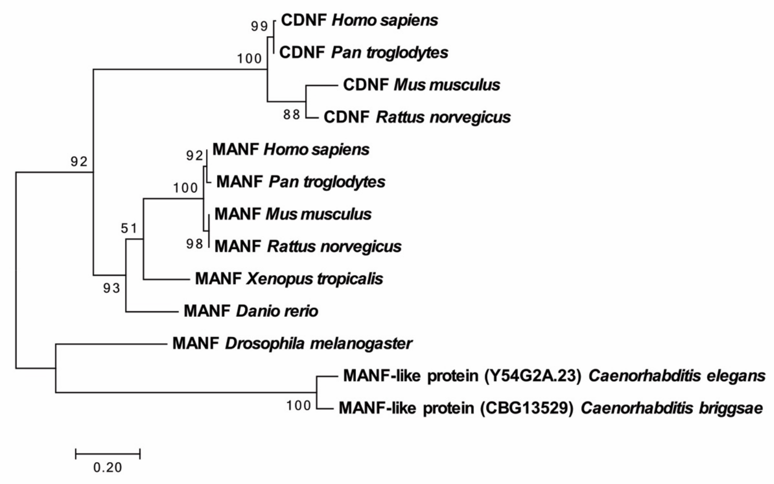

Figure S2. Protein sequence alignments of MANF and CDNF. The signal sequence is depicted in red, eight conserved cysteines in blue, N’ terminal Saponin-like domain is bolded, C’ terminal SAP-domain in orange, C-X-X-C motif in grey and putative ER retention signal in green.

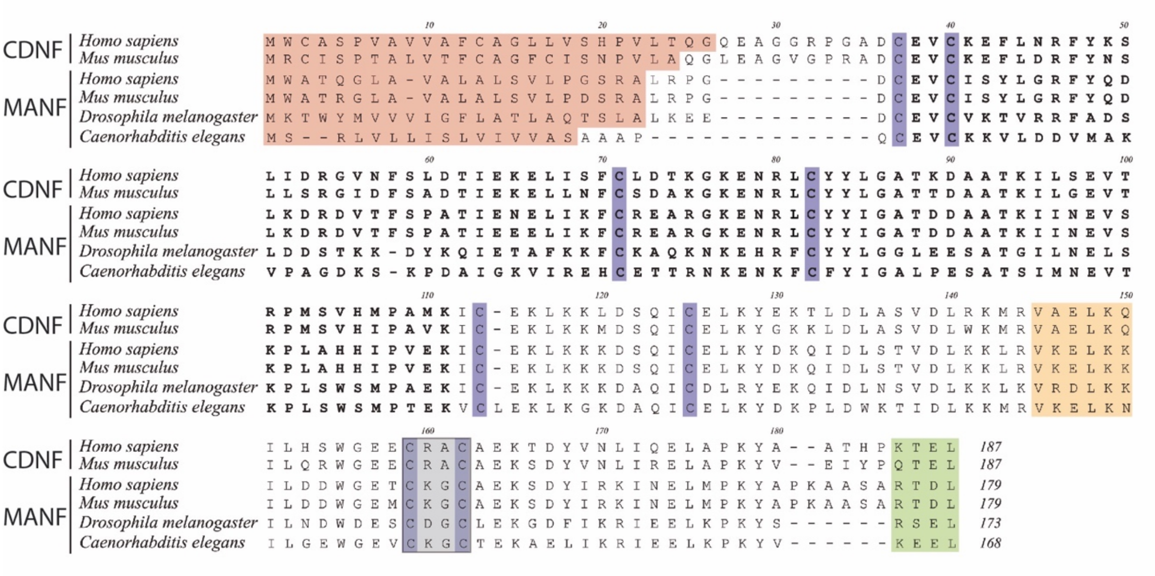

Figure S3. Open reading frame of *manf-1* and deletions in *tm3603* and *gk3677* alleles. The thick black bars represent exons and thin lines represent introns. The arrow marks the 5' to 3' orientation of the gene. The deleted regions in *tm3603* and *gk3677* alleles are depicted by red lines underneath the exons and introns. The nucleotide and amino acid sequences of the truncated transcript generated in *tm3603* animals are shown, indicating the presence of three in-frame nonsense mutations (highlighted).

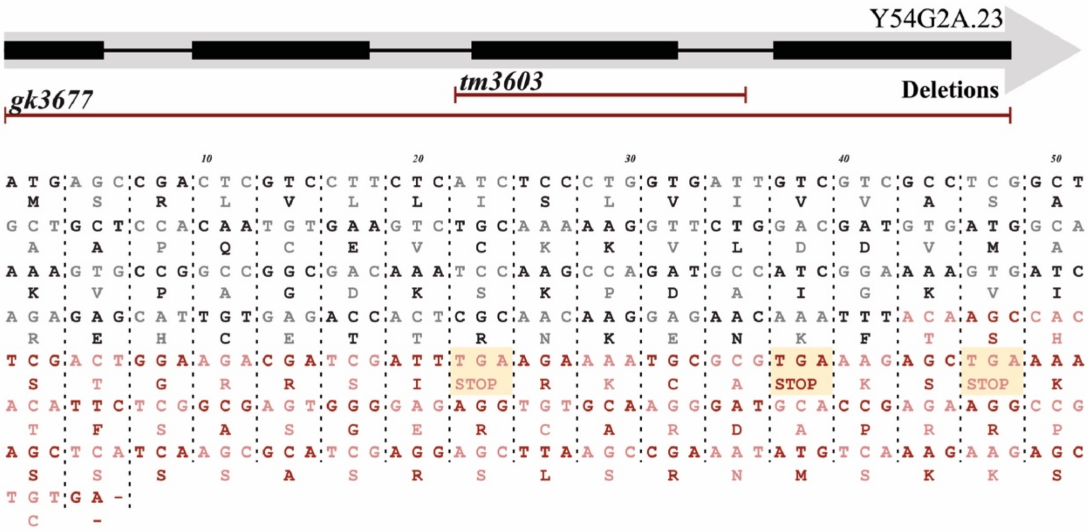

Figure S4. Survival curves of wildtype and *manf-1* mutant animals. Data plotted as the mean values of experiments carried out using two biological replicates, n=120. Graph generated using OASIS 2 software (Han et al., 2016). Mean lifespans: N2 11.76 days and *tm3603* 12.20 days.

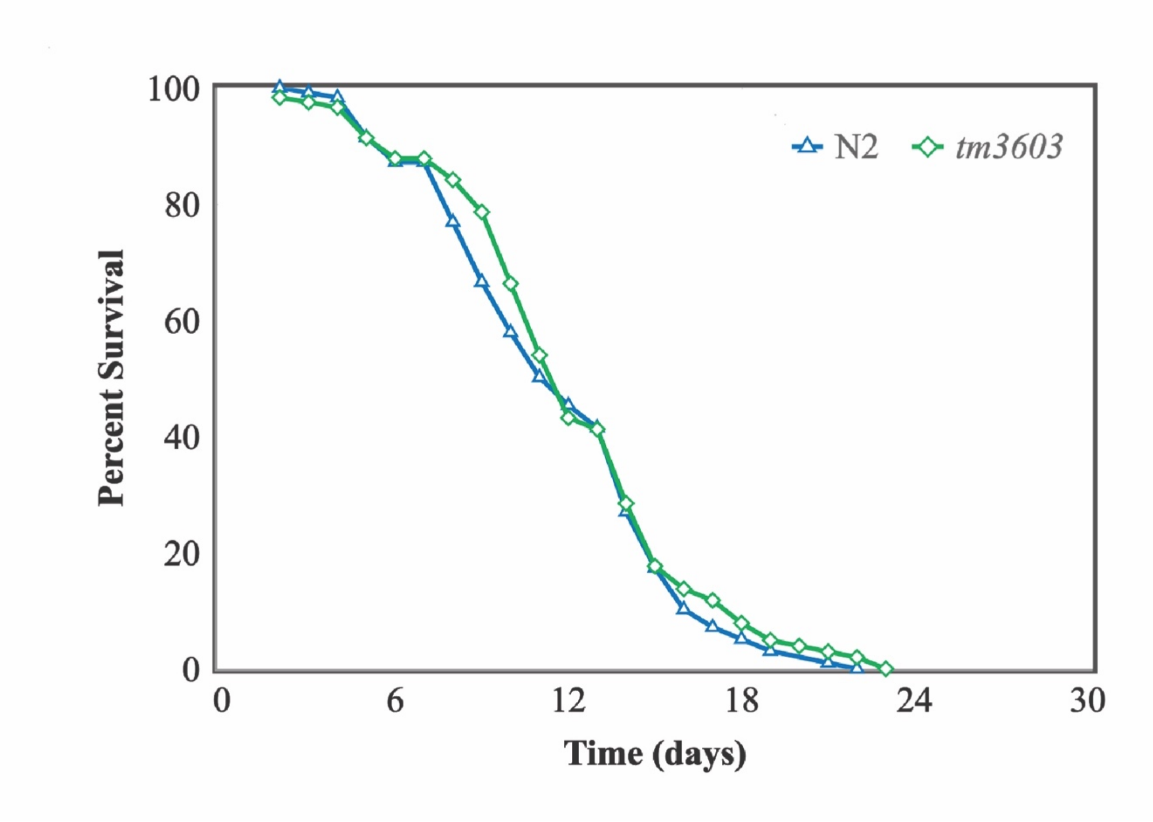

Figure S5. Analysis of GABAergic and serotonergic neurons in *manf-1* mutants. (A-C) Expression analysis of GABAergic *unc-47p*::GFP and (D-F) serotonergic *tph-1p*::GFP neuronal markers in wildtype (WT) and *tm3603* animals. The Neurons are labelled in WT animals, and GABAergic D-type motor neurons are numbered along the ventral cord. **A**nterior, **P**osterior, **D**orsal, **V**entral orientations are marked as appropriate. Arrowheads in D, E and F mark the locations of the vulva. Panels A, B, D, E show day-one adults and C, F day-seven adults. The right hand side images in panels A and B and all three in panel C show representative neurons and commissures at higher magnifications. (A, B) The arrangements of GABAergic neuronal cell bodies and commissure morphologies in WT and *tm3603* day-one adults are normal. (C) Day-seven old animals frequently showed defects such as blob-like appearance next to commissures (marked with star) and split or fused commissures (arrowheads). Dotted white lines mark the outline of worms. In panel F, representative examples of serotonergic neurons in day-seven adults are shown. The phenotypes appear normal in both WT and *tm3603* animals and resemble day-one adults (D and E). In all cases scale bar is equal to 50 μm.

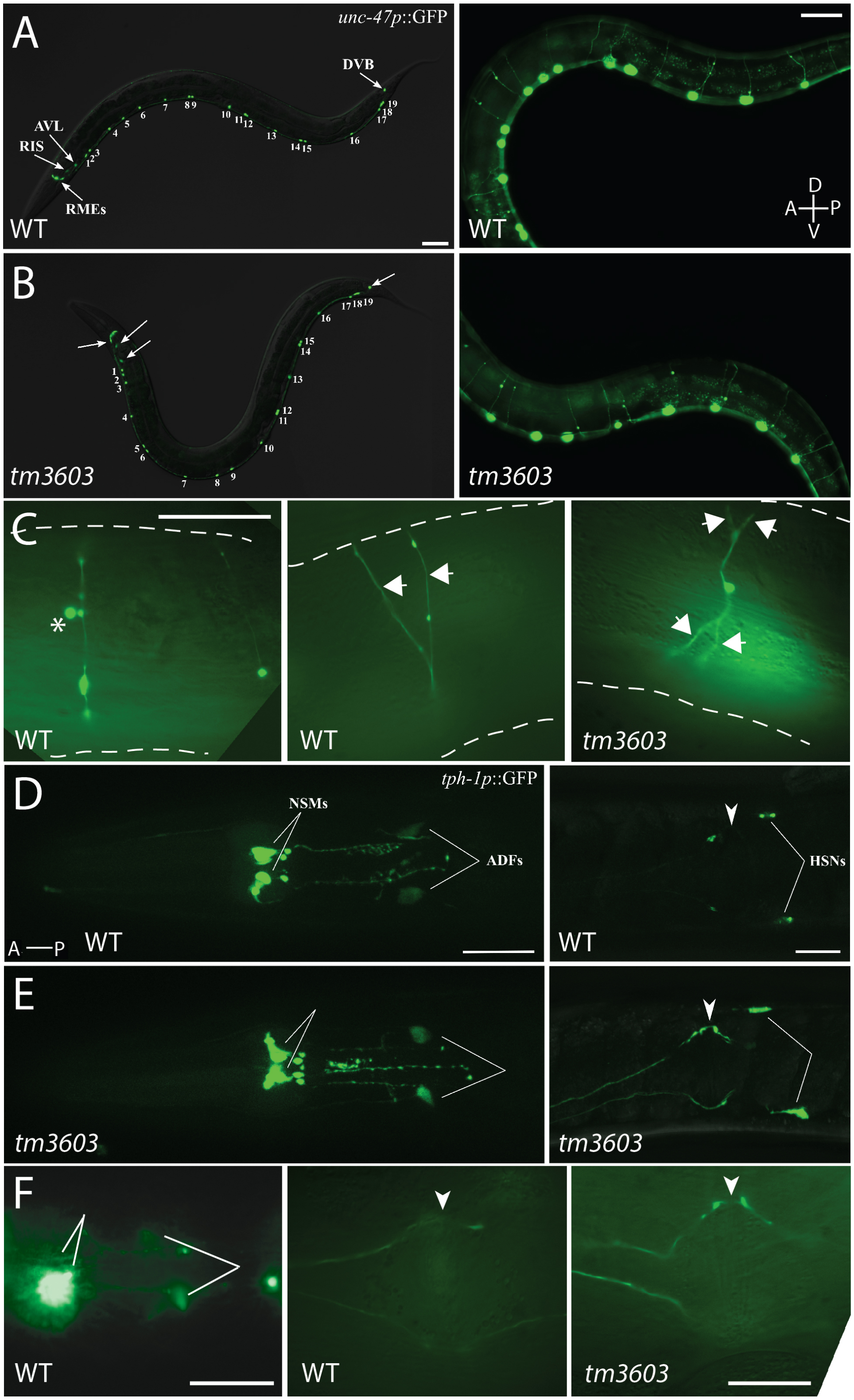
A
unc-47p::GFP
DVB
AVL
RIS
RMEs
WT
D
A
P
V
B
tm3603
C
*
D
tph-1p::GFP
NSMs
ADFs
HSNs
A
P
E
F

Figure S6. ***manf-1*** **transcriptional profile in developmental and adult stages.** (A) Microarray expression analysis during embryonic and L1 larval stages. Transcript levels are shown from the 4-cell embryonic to the first juvenile (L1) larval stages. See *Levin et al.* study for a description of developmental stages and raw data (Levin et al., 2012). (B) Box plot showing age-related changes in *manf-1* expression during adulthood. The plot is based on a published genome-wide microarray expression study (Golden et al., 2008).

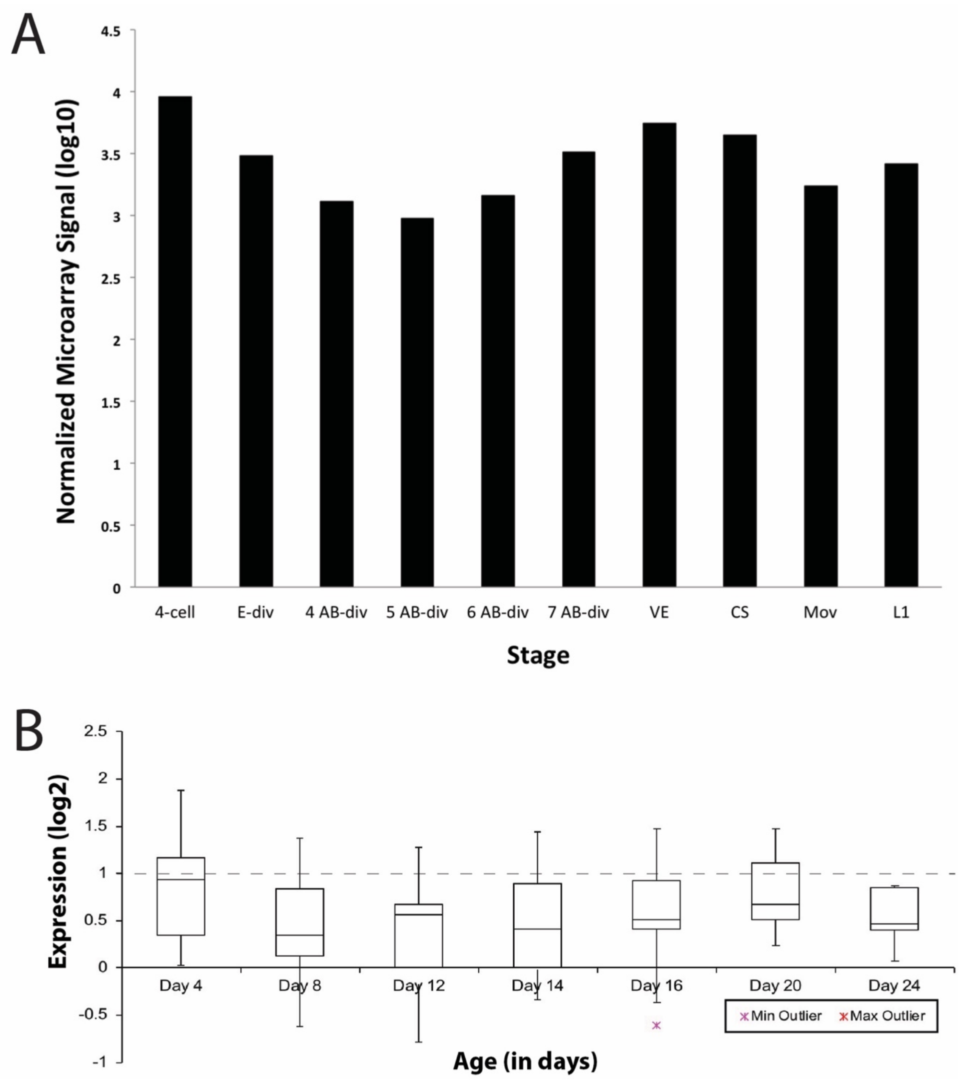

REFERENCES

Golden, T.R., Hubbard, A., Dando, C., Herren, M.A., and Melov, S. (2008). Age-related behaviors have distinct transcriptional profiles in Caenorhabditis elegans. *Aging Cell* 7**,** 850-865.

Han, S.K., Lee, D., Lee, H., Kim, D., Son, H.G., Yang, J.S., Lee, S.V., and Kim, S. (2016). OASIS 2: online application for survival analysis 2 with features for the analysis of maximal lifespan and healthspan in aging research. *Oncotarget* 7**,** 56147-56152.

Kumar, S., Stecher, G., and Tamura, K. (2016). MEGA7: Molecular Evolutionary Genetics Analysis Version 7.0 for Bigger Datasets. *Mol Biol Evol* 33**,** 1870-1874.

Le, S.Q., and Gascuel, O. (2008). An improved general amino acid replacement matrix. *Mol Biol Evol* 25**,** 1307-1320.

Le, S.Q., Lartillot, N., and Gascuel, O. (2008). Phylogenetic mixture models for proteins. *Philos Trans R Soc Lond B Biol Sci* 363**,** 3965-3976.

Levin, M., Hashimshony, T., Wagner, F., and Yanai, I. (2012). Developmental milestones punctuate gene expression in the Caenorhabditis embryo. *Dev Cell* 22**,** 1101-1108.
